# Assessment of nicotine pharmacokinetics and abuse liability in randomized, crossover studies of Vuse Alto electronic nicotine delivery systems

**DOI:** 10.3389/fphar.2026.1796858

**Published:** 2026-07-14

**Authors:** Milly N. Kanobe, Sarah A. Baxter, Kristen Prevette, Tao Jin, Melissa A. Tapia, Sarah Ayoku, Gary M. Dull, Patrudu Makena, John Mohrmann, Brian M. Keyser, Nathan Gale, Kristen G. Jordan

**Affiliations:** 1 RAI Services Company, Winston-Salem, NC, United States; 2 BAT (Investments) Limited, Research and Development, Southampton, United Kingdom

**Keywords:** abuse liability, abuse potential, flavor, nicotine exposure, subjective effects, tobacco harm reduction, Vuse Alto ENDS

## Abstract

**Introduction:**

Understanding the neuropharmacological effects and abuse liability (AL) of electronic nicotine delivery systems (ENDS) is essential for evaluating their potential role in tobacco harm reduction (THR). Nicotine delivered via ENDS engages central nervous system (CNS) pathways involved in reinforcement, craving, and dependence. This research characterized nicotine pharmacokinetics (PK) and AL of Vuse Alto ENDS across multiple nicotine concentrations and flavor and contextualized these findings relative to combustible cigarettes and nicotine replacement therapy (NRT) gum.

**Methods:**

Two randomized, open-label, crossover clinical studies were conducted under controlled human-use conditions. Study 1 evaluated eight Vuse Alto ENDS flavors containing 1.5% nicotine, with sequential plasma sampling over 240 min following product use. Study 2 assessed four Vuse Alto ENDS (1.5%–5% nicotine) and incorporated validated subjective effect assessments and PK sampling, alongside participants’ usual brand cigarettes and NRT gum. Adult smokers and dual users were enrolled under strict eligibility criteria to ensure participant safety and data integrity.

**Results:**

All Vuse Alto variants demonstrated rapid systemic nicotine uptake, with PK profiles consistent across flavors, indicating flavor-independent nicotine delivery. Observed C_max_ ranged from 4.27 to 6.04 ng/mL, AUC_0–15_ from 46.09 to 64.63 ng × min/mL, and AUC_0–240_ from 420 to 509 ng × min/mL. Higher nicotine concentrations produced proportionally greater C_max_ and AUC values, though all remained lower than those from cigarettes. T_max_ for ENDS were consistently 6 min, aligning with the rapid onset typical of inhaled nicotine, while NRT gum exhibited delayed absorption (45 min). Subjective measures including product liking, positive effects, and intent to use again were lower for ENDS compared with cigarettes and generally comparable to NRT gum. Urge-to-smoke decreased within 15 min for all ENDS variants, similar to NRT gum but less rapidly than cigarettes.

**Discussion:**

Findings demonstrate that Vuse Alto ENDS deliver nicotine more slowly and at lower levels than cigarettes, reflecting a lower AL while maintaining sufficient CNS-relevant nicotine exposure to potentially support transitions away from smoking. Flavor did not materially influence PK or AL outcomes. Collectively, the results indicate an intermediate neuropharmacological profile consistent with reduced dependence potential and support the potential utility of Vuse Alto ENDS within THR strategies.

The clinical studies were registered at ClinicalTrials.gov; NCT05239884 and NCT05210699.

## 1 Introduction

Cigarette smoking, reported as the primary cause of preventable deaths in the United States (US), is a major risk factor for serious diseases including lung cancer, chronic obstructive pulmonary and cardiovascular diseases (Centers for Disease Control and Prevention, 2023; U.S. Department of Health and Human Services, 2010). The risk of developing these diseases arises due to prolonged inhalational exposure to the many toxicants generated during the processes of combustion and pyrolysis of the tobacco in cigarettes (Baker et al., 2004; Fowles and Dybing, 2003; Rodgman and Perfetti, 2013). A number of cigarette smoke toxicants have been designated as harmful and potentially harmful constituents (HPHCs) by the US Food and Drug Administration (FDA) and have demonstrated links to cancer, heart disease, lung disease, and reproductive and developmental toxicity (U.S. Food and Drug Administration, 2012). Cigarette smoke also contains high levels of nicotine. Although nicotine is addictive, it is not considered a primary driver of the harmful health effects associated with cigarette smoking (Lee and Fariss, 2016; Newhouse et al., 2012; Murray et al., 2009; Waldum et al., 1996; Murray et al., 1996; Abrams et al., 2018; Gottlieb and Zeller, 2017). The adverse health outcomes of smoking are largely attributable to the complex mixture of toxicants generated during combustion rather than nicotine itself. While some preclinical studies suggest that nicotine may influence certain biological processes (e.g., cell signaling pathways involved in proliferation or angiogenesis) (Singh et al., 2011), these findings do not establish a causal role in disease development, and nicotine is therefore not regarded as a major independent health risk in this context.

A continuum of relative risk is recognized for the use of tobacco and nicotine-containing products, with cigarettes posing the highest health risk and abstinence the lowest. Nicotine-containing medicinal products, such as nicotine replacement therapy (NRT), are associated with minimal disease risk compared to cigarettes. Electronic nicotine delivery systems (ENDS) or e-cigarettes are generally considered to fall between these categories, posing lower risks than combustible cigarettes but higher risks than NRT (Abrams et al., 2018; Hatsukami and Carroll, 2020; Zeller and Hatsukami, 2009; King and Toll, 2023; Fagerström and Eissenberg, 2012; Henningfield and Keenan, 1993; Institute of Medicine, 2001; U.S. Food and Drug Administration, 2025). Although complete abstention from the use of all tobacco products is the best option for minimizing health risks (U.S. Department of Health and Human Services, 2020), of the approximately 30%-50% of smokers who attempt to quit each year, only 7.5% succeed (Pierce, 2022). For those smokers who are unable or unwilling to quit smoking, a tobacco harm reduction (THR) approach has been suggested as beneficial to both individual and population-level health as it proposes that the harm associated with use of the most harmful tobacco products (i.e., cigarettes) can be reduced by smokers switching completely to using alternative tobacco products with lower tobacco-related morbidity and mortality, even though it may not completely eliminate tobacco or nicotine use (Hatsukami and Carroll, 2020; Zeller and Hatsukami, 2009; Institute of Medicine, 2001). Pivotal to THR approaches is the availability of non-combustible tobacco products (such as oral tobacco products and e-cigarettes) to smokers as an alternative to cigarette smoking (Gottlieb and Zeller, 2017; Hatsukami and Carroll, 2020; Institute of Medicine, 2001; Royal College of Physicians, 2016; Health Canada, 2022; New Zealand Ministry of Health, 2020; Office for Health Improvement and Disparities, 2022). Because of the addictive nature of nicotine, the need to reduce the harms associated with smoking while preventing initiation with or addiction to alternative tobacco products has been a topic of debate among the public health community.

Electronic nicotine delivery systems are battery-operated devices that heat nicotine-containing e-liquids to generate an inhalable aerosol (Lauterstein et al., 2020; Williams and Talbot, 2019). ENDS are a highly diverse category of tobacco products (Williams and Talbot, 2019; DeVito and E-cigarettes, 2018) with a significant presence in the US marketplace (Ali et al., 2023a; Ali et al., 2023b). Growing evidence suggests that ENDS may be less harmful than cigarettes, particularly among adult smokers who completely switch from cigarette smoking to exclusive ENDS use (McNeill et al., 2015; National Academies of Sciences, Engineering, and Medicine, 2018; Warner et al., 2023). There is also increasing evidence that ENDS are able to facilitate smoking reductions and cessation (Mok et al., 2022; Auer et al., 2024; Hajek et al., 2019; Hartmann-Boyce et al., 2022; Lindson et al., 2024; Myers Smith et al., 2022; Pope et al., 2024; Przulj et al., 2023; Xu et al., 2024; Carpenter et al., 2023), with an even greater supportive role proposed for flavored ENDS (Yingst et al., 2024; Food and Drug Administration, 2024). However, it is important to note that a substantial proportion of users engage in dual use of ENDS and combustible cigarettes. This pattern is common among tobacco users and has been associated with nicotine and toxicant exposures that are comparable to, or in some cases higher than, those observed with exclusive cigarette smoking, and higher than exclusive ENDS use (Smith et al., 2021; Xue et al., 2025; Dai et al., 2022). Consequently, dual use may attenuate the potential harm reduction benefits associated with complete switching.

Following the passing of the Family Smoking Prevention and Tobacco Control Act in 2009 (United States Congress, 2009), which granted the FDA authority to regulate the manufacture, marketing, and distribution of tobacco products, the FDA now regulates all aspects of tobacco products, including authorizing their sale and marketing, in the U.S.; this authority was further expanded through the FDA’s 2016 “Deeming Rule,” which extended FDA jurisdiction to all product meeting the statutory definition of a tobacco product (U.S. Food and Drug Administration, 2016). Since then, the FDA has issued rules and guidance for manufacturers regarding premarket tobacco product applications (PMTAs) (U.S. Food and Drug Administration, 2021). The predominant feature of this guidance is placing an onus on manufacturers to establish that the marketing of a novel tobacco product is appropriate for the protection of the public health (APPH). Among various assessments recommended to demonstrate that a novel tobacco product is APPH is the assessment of abuse liability (AL) in human clinical studies. Abuse liability influences whether a novel tobacco product is able to completely displace the use of more harmful tobacco products such as cigarettes, as well as whether it poses an addiction and/or initiation risk among tobacco non-users (Carter et al., 2009; U.S. Food and Drug Administration, 2021; Cahn et al., 2021). Abuse liability may be defined as “the likelihood that a drug with psychoactive or central nervous system (CNS) effects will sustain patterns of non-medical self-administration that result in disruptive or undesirable consequences” (Carter and Griffiths, 2009; Balster and Bigelow, 2003). In the context of tobacco products, AL is a term synonymous with ‘dependence potential’ or the ability to induce dependence and/or persistent use and is a composite measure which can be estimated using nicotine pharmacokinetic (PK) and subjective effects assessments (Carter et al., 2009; Fearon, 2022; Vansickel et al., 2022). To support THR, novel, potentially reduced risk tobacco products should exhibit at least some degree of appeal and AL in order to support smokers in switching to their exclusive use, while not having such a high AL to pose an addiction or initiation risk among tobacco non-users (Abrams et al., 2018; Gottlieb and Zeller, 2017; Cahn et al., 2021; Gades et al., 2022; Institute of Medicine, 2012). Regarding ENDS, several studies assessing nicotine PK and subjective effects have suggested that their AL likely lies between cigarettes and NRTs such as nicotine gums and inhalers (Goldenson et al., 2020a; Maloney et al., 2019; Stiles et al., 2017; Stiles et al., 2018; Campbell et al., 2023; Campbell et al., 2022). Vuse is a brand of ENDS that encompasses several generations of ENDS products, ranging from older “cig-a-like” products (e.g., Vuse Solo, Vuse Ciro and Vuse Vibe) to more recent “pod-mod” type ENDS products (e.g., Vuse Alto). Previous studies conducted with these Vuse ENDS products indicate lower levels of non-nicotine HPHCs and toxicants exposure to consumers (Haswell et al., 2023; Kanobe et al., 2022; Kanobe et al., 2023; Liu et al., 2021; Round et al., 2019) and indicate an AL lower than cigarettes but higher than or comparable to FDA -approved NRT gum (Stiles et al., 2017; Stiles et al., 2018; Campbell et al., 2022). Based on the weight of evidence, including lower toxicant emissions and intermediate AL profiles (behavioral and pharmacokinetic) relative to cigarettes, certain Vuse ENDS products have been authorized for marketing by the FDA through the PMTA pathway (U.S. Food and Drug Administration, 2024). Under this framework, the FDA may issue Marketing Granted Orders when a product is deemed to be APPH, taking into account the potential benefits to adult smokers and risks to non-users at the population level. The Vuse Alto device used in the two studies reported here is a “pod-mod” type ENDS that is commercially available in the U.S. While certain Vuse Alto ENDS cartridges were commercially available in the U.S. at the time the studies were conducted, none of the cartridges included in these studies were commercially marketed in the U.S. and were used for research purposes only. The objective of the two clinical studies was to assess the AL potential of Vuse Alto ENDS, thereby providing evidence regarding the potential role of these products in THR approaches.

## 2 Materials and methods

### 2.1 Study design

Nicotine uptake and subjective effects of several Vuse Alto ENDS that differed in nicotine concentrations and flavors were assessed in two independent, randomized, open-label, crossover, confinement clinical studies, termed “Study 1” and “Study 2” (Table 1). Study 1 assessed Vuse Alto ENDS across multiple flavors at a single nicotine concentration (1.5%), assessing plasma nicotine uptake during and following product use, along with overall product liking (OPL). In contrast, Study 2 evaluated nicotine PK and several subjective effects related to reinforcement across multiple nicotine levels within a selected flavor variant, relative to known high- and low- AL comparator products (cigarette and NRT, respectively). Specifically, Study 1 included seven flavored and one unflavored product, each containing 1.5% nicotine, whereas Study 2 assessed two flavored products, one across 1.5%, 2.4%, and 5% nicotine concentrations, and another at 1.5% nicotine, within a broader AL framework that incorporated comparator products. These studies were designed as complementary investigations with distinct primary objectives. Study 1 aimed to evaluate whether flavor influences nicotine PK by assessing multiple flavor variants at a constant nicotine concentration; accordingly, it focused primarily on plasma nicotine endpoints, with limited subjective assessments. In contrast, Study 2 was designed to characterize abuse liability across a smaller subset of flavors tested at multiple nicotine concentrations, enabling assessment of dose-dependent subjective responses and reinforcing effects. The differing scope of subjective measurements between the studies reflects these distinct objectives.

**TABLE 1:** Vuse Alto ENDS and comparator products assessed in Study 1 and Study 2

Study	Product name	Nicotine concentration	Flavor name
Study 1	Vuse Alto ENDS	1.5%	Vuse Alto Menthol
		1.5%	Vuse Alto Rich Tobacco
		1.5%	Vuse Alto Golden Tobacco
		1.5%	Vuse Alto Smooth Tobacco
		1.5%	Vuse Alto Aromatic Tobacco
		1.5%	Vuse Alto Tropical Coconut
		1.5%	Vuse Alto Berry Cream
		1.5%	Vuse Alto Unflavored
Study 2	Vuse Alto ENDS	1.5%	Vuse Alto Menthol
		2.4%	Vuse Alto Menthol
		5%	Vuse Alto Menthol
		1.5%	Vuse Alto Glacier Menthol
	UB cigarette	NA	NA
	NRT gum	4*	White Ice Mint

* nicotine content per piece in milligrams. Abbreviations: ENDS, electronic nicotine delivery system(s); UB, usual brand; NRT, nicotine replacement therapy; NA, not applicable.

Study 1 (ClinicalTrials.gov identifier; date: NCT05239884; 14Mar2022) and Study 2 (ClinicalTrials.gov identifier; date: NCT05210699; 23Jun2022) were conducted between February and June 2022 in the US. The studies were approved by Advarra Institutional Review Board (IRB; Columbia, MD) and were conducted in accordance with the Declaration of Helsinki and applicable sections of US Code of Federal Regulations and ICH E6 Good Clinical Practice standards. All participants provided written informed consent to participate.

**FIGURE 1:**
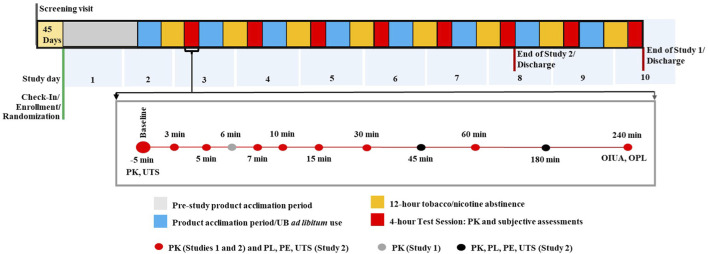
Pharmacokinetics and abuse liability study designs. Nicotine uptake of eight Vuse Alto ENDS (Study 1) was assessed over 10 days, with one study product assessed per day. The AL of four Vuse Alto ENDS (Study 2), along with two comparator products for high (UB combustible cigarette) and low (NRT gum) AL, was assessed over 8 days, with one study product assessed each day. Following check-in/enrollment, participants were randomized and were allowed to acclimatize to the study products. On Days 3–8 (Study 1) and Days 3–10 (Study 2), participants used their randomly-allocated study products during the test sessions. Each test session lasted for 240 min. The expanded timeline shown in the lower portion of the figure illustrates events which occurred during the test sessions. The cycle of product acclimation period, tobacco/nicotine abstentions, and test sessions with assigned study products, were repeated for each test session day. Abbreviations: AL, abuse liability; PL, product liking; PE, product effects; UTS, urge to smoke; OIUA, overall intent to use again; OPL, overall product liking.

Study 1 evaluated eight different Vuse Alto ENDS (Table 1) in separate daily test sessions, with one study product used in each test session within a 10-day confinement period (Figure 1). The aim of this study was to evaluate nicotine uptake and OPL of Vuse Alto ENDS. The primary endpoints focused on determining the maximum plasma nicotine concentration and overall plasma nicotine uptake over 240 min after the start of study product use. Study 2 evaluated six different products including Vuse Alto ENDS, and high- and low-AL comparators, in separate daily test sessions over 8 days of clinical confinement (Table 1; Figure 1). The objective of Study 2 was to evaluate nicotine pharmacokinetics (PK) and subjective effects of Vuse Alto ENDS in comparison to high- and low-AL comparators. The assessment of AL was performed per established methods (Carter et al., 2009; Fearon, 2022; Vansickel et al., 2022) and regulations (U.S. Food and Drug Administration, 2021). Measurements of nicotine PK endpoints in combination with subjective effects constituted the assessment of AL, as described previously (Stiles et al., 2017; Stiles et al., 2018; Campbell et al., 2023; Campbell et al., 2022). The primary endpoints for Study 2 were: 1) the area-under-the-effect curve (AUEC) from 3 min to 240 min after the start of test product use for product liking (PL) (AUEC_PL 3–240_), and 2) the maximum effect for PL after the start of test product use (E_max PL_).

For both studies, on Day 1, participants who met all inclusion criteria and none of the exclusion criteria were enrolled, randomized to a product use sequence based on a Williams Design, and confined at the study site. On Day 1, participants took part in a pre-study Vuse Alto ENDS acclimation period. During this session, participants were required to familiarize themselves with the use of each of the Vuse Alto ENDS (and, in Study 2, one piece of NRT gum) at least once, while having access to their usual brand (UB) cigarettes for *ad libitum* smoking.

Starting on Day 2, and on subsequent days following each morning test session, participants underwent product-specific acclimation periods in which they used their randomized Vuse Alto ENDS (and, in Study 2, a piece of NRT gum) to be tested during the following day’s test session. Participants were permitted to smoke their UB cigarettes *ad libitum* as long as they met the minimum use requirement for the study product used during the acclimation period. Following the acclimation period, participants abstained from the use of all tobacco- and nicotine-containing products for a minimum of 12 h, and from any caffeine-containing products for 240 min prior to and through the end of each test session.

Each test session lasted for a period of 240 min during and following product use. During each test session, participants were allowed 5 min of *ad libitum* use of their assigned Vuse Alto ENDS (and in the case of Study 2, up to 5 min of smoking a single UB cigarette or approximately 30 min of NRT gum use [one piece as per the package labeling]). The participant’s use pattern for UB cigarettes and Vuse Alto ENDS was *ad libitum* and no specific parameters around puff duration, volume, inter-puff interval, and puff quantity were required. Over the course of a test session, blood draws were made for plasma nicotine analyses and responses to subjective effects questionnaires were collected. A schematic of the study designs and procedure timelines can be found in Figure 1.

### 2.2 Study participants

Study 2 recruited exclusive adult smokers of filtered, menthol cigarettes or dual users of both menthol cigarettes and ENDS. Study 1 recruited exclusive adult smokers of menthol or non-menthol, filtered cigarettes or dual users of cigarettes and ENDS. Potential participants were identified by the clinic study site via a database of healthy volunteers and/or advertisements directed at the target population. The main inclusion criteria were male and female adults aged 21–60 years in generally good health, who self-reported primarily smoking at least 10 filtered cigarettes per day for at least 6 months prior to screening, and responded to the Fagerström Test for Nicotine Dependence (FTND) Question 1 (“How soon after you wake up do you smoke your first cigarette?“) with either “Within 5 min” or “6-30 min” (Fagerström and Eissenberg, 2012; Heatherton et al., 1991). Cigarette smoking was confirmed at screening by measuring exhaled carbon monoxide and urine cotinine. Eligibility criteria required an ECO between ≥10 ppm and ≤100 ppm, along with a positive urine cotinine test (≥200 ng/mL) using a dipstick. Females who had a positive pregnancy test, were pregnant, breastfeeding, or intended to become pregnant during the course of the study were excluded from participation.

In both studies, attempts were made to recruit a balance of sexes with no less than 40% of either sex represented, and at least 15%-20% African American participants in an effort to have the study sample reflect the reported percentage of US smokers who are African American (Cornelius et al., 2023).

### 2.3 Study products

Vuse Alto is an ENDS consisting of closed, non-refillable, e-liquid cartridges (also referred to as pods) that work in combination with a non-adjustable power unit with a rechargeable battery (typical capacity 370 mAh). The Vuse Alto ENDS pods contain approximately 1.8 mL of e-liquid of varying flavors and salt-based nicotine contents of 1.5%, 2.4%, and 5.0% by weight. A complete list of the Vuse Alto ENDS variants assessed in Studies, 1 and 2, including their flavors and nicotine concentrations, as well as details regarding the comparators assessed, can be found in Table 1.

Participants supplied their own UB cigarettes for personal use during designated *ad libitum* smoking periods, and for use during the cigarette test session in Study 2. The Vuse Alto ENDS and NRT gum (Nicorette White Ice Mint polacrilex gum, 4 mg nicotine; Glaxo SmithKline, Durham, NC, USA) were provided by the study sponsor.

### 2.4 Study endpoints

#### 2.4.1 Nicotine pharmacokinetics

Nicotine PK parameters for both studies were evaluated as previously described for other Vuse ENDS (Stiles et al., 2017; Stiles et al., 2018; Campbell et al., 2023; Campbell et al., 2022). Blood samples for the assessment of nicotine uptake parameters were collected at predefined time points during each test session, including pre-use (baseline) and multiple post-use time points up to 240 min following product use, to adequately characterize the nicotine concentration-time profile. The nicotine uptake parameters evaluated were maximum plasma nicotine concentration (C_max_), the area under the nicotine concentration-time curve (AUC) from time zero to 240 min after the start of product use (AUC_0-240_), AUC from time zero to 15 min after the start of product use (AUC_0-15_), and the time to reach C_max_ (T_max_).

Blood samples were collected at predefined time points, including pre-use (baseline) and multiple post-use time points up to 240 min following product use, to characterize the nicotine concentration–time profile. Sampling was conducted via an indwelling intravenous catheter placed by qualified clinical personnel (e.g., a registered nurse). A single catheter was typically maintained per study day to minimize repeated venipunctures, with site rotation performed as appropriate to reduce local irritation and maintain vein condition across the study period.

The total blood volume collected per participant over the course of the study was approximately 505 mL (Study 1) and approximately 467 mL (Study 2), including both clinical laboratory assessments and PK sampling. This volume is comparable to that obtained during a standard whole blood donation by the American Red Cross (approximately 500 mL) in a single sitting. Participants were monitored for signs or symptoms related to blood collection (e.g., lightheadedness, bruising, or bleeding), with appropriate management provided as needed. Given the total volume collected, participants were advised to refrain from donating blood or plasma for at least 7 days following study completion.

#### 2.4.2 Subjective effects measures

The only subjective effects measure assessed in Study 1 was OPL at the 240 min time point (Figure 1). Study 2 evaluated five subjective effects measures through responses to questionnaires administered at various time points during the test session. Each questionnaire asked a single question with responses on a numerical rating scale (NRS; 0, “Not at all”; 10, “Very much”) (Stiles et al., 2017; Stiles et al., 2018; Campbell et al., 2022). The subjective effects measures and their respective parameters were defined previously (Stiles et al., 2017; Stiles et al., 2018; Campbell et al., 2022) and included PL, urge to smoke (UTS), and product effects (PE; both positive and negative PE), which were assessed at several nominal timepoints over the 240 min following the start of product use; the UTS questionnaire was also administered 5 min prior to the start of product use (Figure 1). Additionally, at the 240-min time point, participants were asked to assess their overall intent to use (*the product*) again (OIUA) and OPL.

Study 2 assessed PL, a subjective measure of positive reinforcement, over the 240 min of the test session following the start of product use. The endpoints for PL were the effect of maximum PL (E_max PL_) and the AUEC for PL score versus time curve from 3 min to 240 min after the start of product use (AUEC_PL 3__–__240_). Additional parameters for UTS, or negative reinforcement, (AUEC for UTS from 0 to 15 min [AUEC_UTS 0__–__15_] and from 0 to 240 min [AUEC_UTS 0__–__240_]), and minimum UTS score after the start of product use [E_min UTS_]), OPL (effect of overall PL [E_overall PL_]), PE (AUEC for positive or rewarding PE from 3 to 240 min [AUEC_PEpos 3__–__240_], maximum effect of positive PE [E_max PEpos_], AUEC for negative PE (a subjective measure of avoidance behavior) from 3 to 240 min [AUEC_PEneg 3__–__240_], and maximum effect of negative PE [E_max PEneg_]), and OIUA (effect of overall OIUA [E_overall IUA_]) were also evaluated.

### 2.5 Safety and adverse events assessment

Participant safety and adverse events (AEs) were monitored and assessed throughout the study duration via medical history, physical and oral examinations, vital sign measurements, and clinical laboratory tests (including hematology, clinical chemistry, and urinalysis). An AE was defined as any untoward medical occurrence or condition experienced by a participant after signing the Informed Consent Form (ICF) until completion of the study, whether or not considered related to the use of the study product. The occurrence of any AE was coded by primary system organ class and preferred term according to the Medical Dictionary for Regulatory Activities Version 24.1 (MedDRA Maintenance and Support Services Organization, 2021). The severity of an AE was categorized as mild, moderate, or severe and the relationship of AEs with product use (not related, unlikely related, possibly related, and related) was classified by the Principal Investigator (PI).

### 2.6 Sample size calculations

For Study 1, the sample size determination was based on the goal of achieving a 95% confidence interval with a half-width that is within 20% of the primary endpoint mean values based on data from a previous study (Campbell et al., 2022). The target number of randomized participants for this study was approximately 40 participants. This allowed for approximately a 20% dropout rate (eight participants) with a goal of 32 participants completing the study.

The sample size for Study 2 was estimated using data from previous ENDS AL studies (Stiles et al., 2017; Stiles et al., 2018; Campbell et al., 2022). Based on those studies, at least 42 participants should be randomized to ensure that at least 36 participants complete the study, providing 80% power to detect the hypothesized differences in each primary endpoint between each Vuse Alto ENDS and each comparator product at a two-sided 0.0031 significance level. This significance level was Bonferroni-adjusted to preserve an overall statistical significance of 0.05 across multiple comparisons. The anticipated dropout rate was approximately 14%.

### 2.7 Statistical analyses

For both studies, individual plasma nicotine concentrations were baseline-adjusted using a model which assumed that nicotine elimination followed first order kinetics (Campbell et al., 2022; Shiffman et al., 2009); this model was used in calculating the PK parameters (AUC_0-15_, AUC_0-240_, C_max_, and T_max_).

For Study 1, the primary PK parameters and OPL were summarized using descriptive statistics and no between-group comparisons were performed.

For analysis of the primary subjective effects in Study 1, scores for AUEC_PL 3–240_ and E_max PL_ for each Vuse Alto ENDS were compared to those for both the high and low AL comparators (UB cigarette and NRT gum, respectively) by using a mixed-effects analysis of variance (ANOVA) model with Bonferroni correction for multiple comparisons using a significance level of 0.0031. For secondary endpoints including AUEC_PEpos 3–240_, E_max PEpos_, AUEC_PEneg 3–240_, E_max PEneg_, E_overall PL_, and E_overall IUA_ for each Vuse Alto ENDS were compared to those for both the high- and low-AL comparators by an ANOVA. Scores for all UTS measures (AUEC_UTS 0–15_, AUEC_UTS 0–240_, and E_min UTS_ for each Vuse Alto ENDS and the high- and low-AL comparators were calculated and compared using a mixed-effects analysis of covariance. Nicotine PK parameters for each Vuse Alto ENDS and the high and low AL comparators were compared using a mixed-effects model ANOVA. A Wilcoxon nonparametric signed rank test was used for comparisons of T_max_ between each Vuse Alto ENDS and the high- and low-AL comparators. For all secondary subjective measure and nicotine PK endpoints, a *P* level of equal to or less than 0.05 was considered significant.

Pharmacokinetic parameters and subjective effects measures were calculated using a SAS® program which followed the same algorithm of noncompartmental analysis (NCA) as that implemented in WinNonlin® Professional (Version 8.0, Pharsight Corporation, A Certara Company, St. Louis, MO). SAS results were validated using the parameters generated from WinNonlin version 8.0.

## 3 Results

### 3.1 Disposition of study participants in studies 1 and 2

Participant disposition is summarized in Supplementary Table S1. Study 1 enrolled 38 participants, with 36 (95%) participants completing the study. Two participants were withdrawn from the study: one participant was discontinued from the study due to study protocol non-compliance, and the other participant was discontinued at the discretion of the PI. The participant withdrawn at the PI’s discretion had a pre-existing food-related allergy (onion allergy) that could potentially have impacted study compliance.

Of the 43 participants enrolled in Study 2, 42 (97.7%) participants completed the study, and one participant withdrew from the study as they could not tolerate intravenous blood draws due to poor vein access.

### 3.2 Demographics and baseline characteristics of the study participants

A summary of demographics and baseline characteristics for participants is presented in Supplementary Table S2. In Study 1, 74% of participants were male, 8% were Hispanic/Latino, 55% were Black/African American, and 3% were of native Hawaiian/other Pacific Island origin. In Study 2, 67% of participants were male, all were non-Hispanic/Latino, 27.9% were Black/African American, and 2.3% were of native Hawaiian/other Pacific Island origin. In terms of age and other baseline characteristics, participant populations between both Studies 1 and 2 were similar (Supplementary Table S2). In addition, there were no major differences in participant demographics and baseline characteristics between each randomization sequence group in either Study 1 or Study 2 (data not shown).

### 3.3 Nicotine pharmacokinetics

**FIGURE 2:**
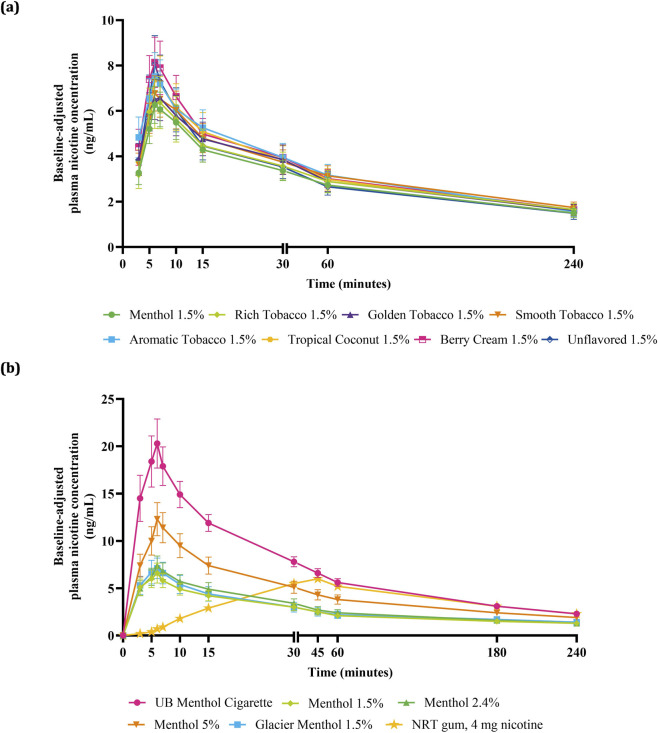
Baseline-adjusted mean plasma nicotine concentration profiles over time for Vuse Alto ENDS and comparator products. Data are presented from **(a)** Study 1 and **(b)** Study 2 as means ± SEM. Abbreviations: SEM, standard error of the mean; ENDS, electronic nicotine delivery system(s); UB, usual brand; NRT, nicotine replacement therapy.

In Study 1, the mean nicotine uptake profiles for each Vuse Alto ENDS assessed were generally similar to each other (Figure 2a). For all Vuse Alto ENDS, plasma nicotine concentrations rose rapidly within 3 min of product use initiation, peaked at approximately 6–7 min, and declined thereafter. The mean plasma nicotine concentration, however, stayed above the baseline through the course of the 240-min test session. Baseline-adjusted nicotine PK parameters (C_max_, AUC_0-15_, AUC_0-240_) and T_max_ are presented in Table 2. The least squares (LS) mean C_max_ ranged from 4.27 ng/mL (Rich Tobacco) to 6.04 ng/mL (Berry Cream). Nicotine uptake during the first 15 min of product use (AUC_0-15_) for the eight Vuse Alto ENDS was between 46.09 ng x min/mL (Golden Tobacco) and 64.63 ng x min/mL (Berry Cream). Total nicotine uptake up to the 240-min time point (AUC_0-240_) was between 420 ng x min/mL (Golden Tobacco) and 509 ng x min/mL (Berry Cream). The median T_max_ values for Vuse Alto ENDS were similar and ranged from 6 to 7 min following start of product use, consistent with the product use period of 5 min (Table 2).

**TABLE 2:** Plasma nicotine pharmacokinetic parameters of Vuse Alto ENDS in Study 1 and Study 2

​	Study 1 Vuse Alto ENDS (1.5% nicotine concentration)								Study 2 Vuse Alto ENDS and comparator products					
Parameter*	Menthol	Rich Tobacco	Golden Tobacco	Smooth Tobacco	Aromatic Tobacco	Tropical Coconut	Berry Cream	Unflavored	Menthol (1.5%)	Menthol (2.4%)	Menthol (5%)	Glacier Menthol (1.5%)	UB cigarette	NRT gum
N	32	31	34	33	32	32	31	31	36	33	33	35	34	33
C_max_ (ng/mL)	4.8	4.3	4.7	5.5	5.4	5.2	6.0	5.2	5.3^a^	6.3^a^	9.5^a^ ^,^ ^b^	5.5^a^	20.2	5.9
AUC_0-15_ (ng x min/mL)	49.7	47.5	46.1	53.0	49.0	53.6	64.6	54.7	50.3^a^ ^,^ ^b^	58.6^a^ ^,^ ^b^	87.1^a^ ^,^ ^b^	52.1^a^ ^,^ ^b^	193.0	11.4
AUC_0-240_ (ng x min/mL)	437.1	434.2	420.0	474.6	446.0	449.8	509.1	437.2	398.0^a^ ^,^ ^b^	447.0^a^ ^,^ ^b^	658.0^a^	402.0^a^ ^,^ ^b^	1265.0	805.0
T_max_ (minutes)	6.0	7.0	7.0	6.0	6.0	6.7	6.1	6.0	6.0^b^	6.0^b^	6.0^b^	6.0^b^	6.0	45.0

* Least squares (LS) means are presented for C_max_, AUC_0-15_ and AUC_0-240_ parameters, and median values are presented for T_max_. Percentages in parentheses are the Vuse Alto ENDS, nicotine concentrations.

^a^ Significantly different (p < 0.05) from UB, cigarettes.

^b^ Significantly different (p < 0.05) from NRT, gum. Abbreviations: N, number of participants; C_max_, maximum plasma nicotine concentration; AUC_0-15_, area under the plasma nicotine concentration-time curve from time zero to 15 min after the start of product use; AUC_0-240_, area under the plasma nicotine concentration-time curve from time zero to 240 min after the start of product use; T_max_, time to reach the maximum plasma nicotine concentration; ENDS, electronic nicotine delivery system(s); UB, usual brand.

In Study 2, the nicotine uptake profiles of the Vuse Alto ENDS and the UB menthol cigarette, although different in magnitude, were generally similar, with a rapid rise in mean plasma nicotine concentration followed by a sustained decline which remained above the baseline through the course of the test session (Figure 2b). The use of NRT gum, however, resulted in a slower increase in plasma nicotine concentration (Figure 2b). Notably, the profile and magnitude of the rise in plasma nicotine concentrations was similar for the 1.5% nicotine concentration Vuse Alto ENDS that were assessed in both Studies 1 and 2 (Figures 2a,b).

The LS mean nicotine PK parameters C_max_, AUC_0-15_, and AUC_0-240_ were higher for the UB cigarette than for all of the Vuse Alto ENDS and NRT gum (Table 2). Least squares mean C_max_ for the UB cigarette was 20.2 ng/mL, ranged from 5.33 ng/mL (Menthol 1.5%) to 9.49 ng/mL (Menthol 5%) for Vuse Alto ENDS, and was 5.89 ng/mL for NRT gum. The differences in C_max_ between the UB cigarette and all Vuse Alto ENDS, and between Vuse Alto Menthol 5% and NRT gum, were significant (Table 2). Nicotine uptake at 15 (AUC_0-15_) and over 240 (AUC_0-240_) minutes after product use was 193 ng x min/mL and 1,265 ng x min/mL, respectively, for the UB cigarette. Among the Vuse Alto ENDS, these AUC parameters were lowest for Menthol 1.5% (50.3 ng x min/mL and 398 ng x min/mL, respectively) and highest for Menthol 5% (587.1 ng x min/mL and 658 ng x min/mL, respectively). Differences in both these AUC parameters were significant between the UB cigarette and all of the Vuse Alto ENDS (Table 2). The AUC_0-15_ and AUC_0-240_ values for NRT gum were 11.4 ng x min/mL and 805 ng x min/mL, respectively, reflecting the slower uptake of nicotine from the NRT gum during use. The AUC_0-15_ and AUC_0-240_ values were significantly different for all of the Vuse Alto ENDS compared to NRT gum, apart from Menthol 5%, which was not significantly different compared to NRT gum (Table 2). Median T_max_ values were the same (6 min) for each Vuse Alto ENDS and the UB cigarette, while median T_max_ for the NRT gum (45 min) was significantly longer than all of the Vuse Alto ENDS (Table 2). These differences are consistent with both the different use periods and the distinct routes of administration; with inhalation (ENDS and cigarettes) resulting in more rapid nicotine absorption compared with the buccal absorption from NRT gum.

### 3.4 Subjective effects measures

In Study 1, a single subjective effects measure, OPL, was evaluated for each of the eight Vuse Alto ENDS, each in 1.5% nicotine concentration (Table 3). Mean (SD) E_overall PL_ scores ranged from 3.7 (±3.03) for Rich Tobacco to 7.7 (±2.47) for Berry Cream. The E_overall PL_ score for the unflavored Vuse Alto ENDS was 5.4 (±2.41).

**TABLE 3:** Overall product liking scores for Vuse Alto ENDS flavor variants in Study 1

Vuse Alto ENDS (1.5% nicotine concentration)	E_overall PL_ Mean (SD)
Menthol [N = 37]	6.0 (2.8)
Rich Tobacco [N = 37]	3.7 (3.0)
Golden Tobacco [N = 37]	4.4 (2.8)
Smooth Tobacco [N = 37]	5.9 (2.4)
Aromatic Tobacco [N = 36]	6.4 (2.4)
Tropical Coconut [N = 36]	7.2 (2.4)
Berry Cream [N = 36]	7.7 (2.5)
Unflavored [N = 36]	5.4 (2.3)

Abbreviations: ENDS, electronic nicotine delivery system(s); E_overall PL_, effect of overall product liking; SD, standard deviation; N, number of participants.

**FIGURE 3:**
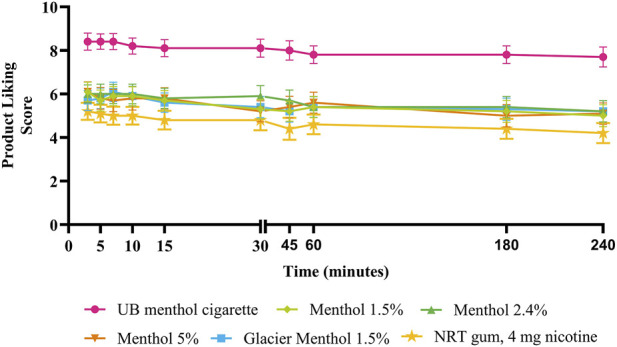
Time course of product liking over time in Study 2. Product liking (PL) scores for Vuse Alto ENDS and comparator products are presented as the maximum PL NRS score (E_max PL_) over time. Data are presented as means ± SEM. Product liking scores were generated by analysis of participants’ responses on a NRS ranging from 0–10 (Stiles et al., 2017; Stiles et al., 2018; Campbell et al., 2022), with 10 representing the highest possible PL. Abbreviations: NRS, numerical rating scale; E_max PL,_ the maximum effect for PL after the start of test product use; SEM, standard error of the mean; ENDS, electronic nicotine delivery system(s).

Study 2 assessed five subjective effects measures for each of the four Vuse Alto ENDS and the high and low AL comparators. The primary endpoint (PL) was assessed using two measures: AUEC_PL 3–240_ and E_max PL_. Among the study products, UB cigarette had the highest PL scores (Figure 3; Table 4), as evident by a geometric least squares (GLS) mean AUEC_PL 3–240_ score of 1806 and an E_max PL_ of 9.02, whereas the NRT gum had the lowest GLS mean scores for these two parameters, 1,044 and 5.83, respectively (Table 4). Product liking parameters, as reflected by the E_max PL_, were highest for UB cigarettes and did not change throughout the course of the test sessions, whereas for each Vuse Alto ENDS, they were lower than UB cigarettes, similar to one another, and remained relatively unchanged over the session time course (Figure 3). The GLS mean AUEC_PL 3–240_ scores for the four Vuse Alto ENDS (Menthol 1.5%, 2.4%, and 5% and Glacier Menthol 1.5%) ranged from 1,242 to 1,300, and values for all Vuse Alto ENDS were significantly lower than UB cigarette (Table 4). The E_max PL_ was comparable among the four Vuse Alto ENDS (6.72–7.03) and was significantly lower than UB cigarette. However, both PL measures for all the Vuse Alto ENDS were not significantly different from NRT gum (Table 4).

**TABLE 4:** Assessment of subjective effects for Vuse Alto ENDS and comparator products in Study 2

Subjective effects parameter*	Menthol (1.5%)	Menthol (2.4%)	Menthol (5%)	Glacier Menthol (1.5%)	UB cigarette	NRT gum
Product liking (AUEC_PL 3–240_)	1264.0^a^	1300.0^a^	1242.0^a^	1263.0^a^	1806.0	1044.0
Product liking (E_max PL_)	6.8^a^	7.0^a^	6.9^a^	6.7^a^	9.0	5.8
UTS (AUEC_UTS 0–15_)	85.3^a^	85.8^a^	82.4^a^	87.9^a^	57.2	87.8
UTS (AUEC_UTS 0–240_)	1751.0^a^	1801.0^a^	1774.0^a^	1830.0^a^	1610.0	1811.0
UTS (E_min UTS_)	5.2^a^	5.2^a^ ^,^ ^b^	4.9^a^ ^,^ ^b^	5.5^a^	3.3	6.0
Positive PE (AUEC_PEpos 3–240_)	893.0^a^	887.0^a^	929.0^a^	856.0^a^	1185.0	871.0
Positive PE (E_max PEpos_)	6.8^a^ ^,^ ^b^	6.8^a^ ^,^ ^b^	6.9^a^ ^,^ ^b^	6.2^a^	9.1	5.8
Negative PE (AUEC_PEneg 3–240_)	264.0	362.0	385.0	345.0	298.0	344.0
Negative PE (E_max PEneg_)	2.7	2.7	3.0	2.6	3.0	2.8
OPL (E_overall PL_)	5.6^a^	5.7^a^	5.8^a^ ^,^ ^b^	5.6^a^	8.2	4.9
OIUA (E_overall IUA_)	4.3^a^	5.3^a^ ^,^ ^b^	5.3^a^ ^,^ ^b^	5.4^a^ ^,^ ^b^	8.0	3.6

* Geometric Least Squares (GLS) means are presented for the parameters. Percentages in parentheses are the Vuse Alto ENDS, nicotine concentrations.

^a^ Significantly different (p < 0.0031 for PL, and p < 0.05 for all other parameters) from usual brand (UB) cigarettes.

^b^ Significantly different (p < 0.05) from NRT, gum. Abbreviations: PL, product liking; ENDS, electronic nicotine delivery system(s); UB, usual brand; NRT, nicotine replacement therapy; UTS, urge to smoke; PE, product effects; OPL, overall product liking; OIUA, overall intent to use again; AUEC_PL, 3–240_, area under the effect curve for PL, between 3 and 240 min; E_max PL_, effect of maximum PL; AUEC_UTS_, _0–15_, area under the effect curve for UTS, between 0 and 15 min; AUEC_UTS, 0–240_, area under the effect curve for UTS, between 0 and 240 min; E_min UTS_, minimum UTS, score after the start of product use; AUEC_PEpos, 3–240_, area under the effect curve for positive PE, between 3 and 240 min; E_max PEpos_, maximum effect of positive PE; AUEC_PEneg, 3–240_, area under the effect curve for negative PE, between 3 and 240 min; E_max PEneg_, effect of maximum negative PE; E_overall PL_, effect of OPL; E_overall IUA_, effect of OIUA.

**FIGURE 4:**
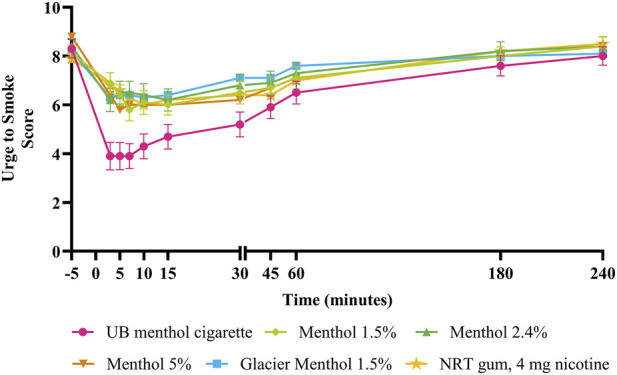
Time course of changes in urge to smoke (UTS) in Study 2. Urge to smoke scores for Vuse Alto ENDS and comparator products were determined from participants’ responses to a single-item questionnaire administered at various time points. Data are presented as means ± SEM. Abbreviations: SEM, standard error of the mean; ENDS, electronic nicotine delivery system(s).

Study2 assessed additional subjective endpoints including UTS, PE, OPL, and OIUA, at different timepoints during the test session. All endpoints assessed for Vuse Alto ENDS were significantly different from the UB cigarettes, with the exception of the parameters for negative PE (Table 4). UB cigarettes were most effective among the study products in relieving smoking urges (Figure 4). The evaluation of AUEC_UTS__0–15_ indicated that UB cigarette was able to reduce UTS within the first 15 min of product use to a significantly greater extent compared to each Vuse Alto ENDS. Overall UTS (AUEC_UTS 0–240_) was significantly greater for each Vuse Alto ENDS compared to UB cigarette. UTS parameters (AUEC_UTS 0–15_, AUEC_UTS 0–240_, and E_min UTS_) for Vuse Alto ENDS were not significantly different compared to NRT gum, with the exception of E_min UTS_ which was significantly lower for Menthol 2.4% and 5% compared to NRT gum (Table 4).

The PE measures, AUEC_3-240_ and E_max PE_, were assessed for both positive and negative PE, and E_overall PL_ and E_overall IUA_ were assessed for OPL and OIUA, respectively, with results significantly lower for each Vuse Alto ENDS compared to UB cigarette (Table 4).

The AUEC_PEpos 3–240_ with use of Vuse Alto ENDS was not significantly different compared to NRT gum, while the E_max PEpos_ was significantly higher compared to NRT gum, except for Glacier Menthol 1.5%, which was comparable to NRT gum. Both overall and maximum negative PE parameter scores (AUEC_PEneg 3–240_ and E_max PEneg_, respectively) for each Vuse Alto ENDS were comparable to each of the two AL comparators.

The OPL scores were highest (8.24) for UB cigarettes, and lowest for NRT gum (4.85), with the Vuse Alto ENDS, ranging from 5.59 to 5.89. Overall PL for Menthol 1.5% and 2.4%, and Glacier Menthol 1.5% was comparable to NRT gum but was significantly higher for Menthol 5% compared to NRT gum.

The OIUA parameter, E_overall IUA_, was highest (8.04) for UB cigarettes, and lowest for NRT gum (3.55). The OIUA scores for all Vuse Alto ENDS ranged from 4.32 (Menthol 1.5%) to 5.44 (Glacier Menthol 1.5%) and were significantly lower than UB cigarettes. The E_overall IUA_ was significantly higher for all Vuse Alto ENDS compared to NRT gum, with the exception of the Menthol 1.5% ENDS which was not significantly different (Table 4).

### 3.5 Adverse events in the study populations

The AEs reported in Studies 1 and 2 are summarized in Supplementary Tables S3-S6. There were no serious adverse events (SAEs) and no AEs led to discontinuation of study product use or termination from Study 1 or Study 2. All AEs were resolved without medication. In Study 1, a total of 3 AEs were reported by three participants (Supplementary Table S3), including conjunctival irritation that was present at study check-in, vessel puncture site pain (Berry Cream, 1.5%) and musculoskeletal pain (Unflavored, 1.5%). These events were all mild in severity and the two reported during the test sessions were considered not related to study product by the PI (Supplementary Table S4).

In Study 2, nine participants reported at least one AE for a total of 17 AEs over the course of the study (Supplementary Table S4; Supplementary Table S6). One of the reported AEs started after signing the ICF and before check-in on Day 1. Nine participants reported an AE during the product acclimation period and/or test session days. The majority (16/17) of these AEs were mild in severity and one was moderate. These AEs were reported by participants while using Menthol 1.5%, Glacier Menthol 1.5%, UB cigarette, and NRT gum. Overall, 3 participants experienced AEs (dizziness and wheezing for Menthol 1.5% nicotine, and throat irritation following NRT gum use) which were causally attributed to study product use by the PI (Supplementary Table S6).

## 4 Discussion

The overall aim of the clinical studies described in this paper was to assess the PK and AL of a range of Vuse Alto ENDS that varied in flavor and nicotine concentration. The AL of tobacco products is a key determinant of initiation, including switching behaviors among smokers who initiate use (Carter et al., 2009; U.S. Food and Drug Administration, 2021), and therefore potential exposure to toxicants and risk of tobacco-related disease. Studies such as those presented here provide valuable information on nicotine uptake and AL under controlled conditions, although their implications for population-level health risk requires consideration alongside real-world use patterns and broader evidence. Key findings from clinical Studies 1 and 2 were: 1) the nicotine uptake profiles of Vuse Alto ENDS were very similar, independent of the ENDS flavor, and dependent on the nicotine concentration; and 2) AL, when assessed as a composite measure informed by nicotine PK and subjective effects measures (Carter et al., 2009; Fearon, 2022; Vansickel et al., 2022), of Vuse Alto ENDS, is significantly lower than the high AL comparator, UB cigarettes, and generally comparable to or greater than the low AL comparator, NRT gum. Our findings are in agreement with other, previous studies with earlier generations of Vuse ENDS (Vuse Solo (Stiles et al., 2017; Stiles et al., 2018; Campbell et al., 2022), Vuse Vibe, and Vuse Ciro (Campbell et al., 2023)), which also revealed a lower AL of the ENDS than UB cigarettes and a somewhat greater, or similar, AL compared to NRT gum. In addition, our findings concur with an earlier assessment of the AL of an earlier generation Vuse Alto 5% ENDS which reported an AL intermediate to cigarettes and NRT gum (Goldenson et al., 2020a). Similar AL findings have also been reported for other pod-system ENDS such as JUUL (Goldenson et al., 2020a).

Across the spectrum of tobacco and nicotine products, cigarettes exhibit the fastest nicotine delivery, the highest uptake of nicotine, are most satisfying to smokers, and therefore possess the highest AL (Maloney et al., 2019; Stiles et al., 2018; Campbell et al., 2023; Fearon et al., 2022; Goldenson et al., 2020b). At the other end of the spectrum, NRTs including gum and other orally administered forms of NRT deliver nicotine more slowly and are less satisfying and have a lower AL (Henningfield and Keenan, 1993; Benowitz, 2009; Benowitz et al., 2021). This is important, since presenting at least some degree of AL is thought to be important in making novel tobacco products more acceptable as alternatives to cigarette smoking, to facilitate persistent and complete displacement of cigarettes among smokers, and to contribute to THR (Abrams et al., 2018; Gades et al., 2022; Ashley et al., 2020). However, too high an AL may give rise to an initiation and/or addiction risk of a novel tobacco product (Cahn et al., 2021; Ashley et al., 2020). Findings from Study 2 reported here showed that participants’ UB cigarettes provided rapid, and the highest degree of, nicotine delivery, which, in addition to the subjective effects measures findings, attest to their high AL (Carter et al., 2009; Fearon, 2022; Vansickel et al., 2022). In contrast, use of NRT gum resulted in a slower and lower uptake of nicotine, which along with the subjective effects assessments confirmed a comparatively low AL of NRT gum. Nicotine delivery from the Vuse Alto ENDS in Study 2 was similar in the rate of onset (T_max_) but significantly lower in magnitude (C_max_) than that from UB cigarettes, and subjective effects such as satisfaction and liking were also lower. Compared with NRT gum, nicotine delivery from Vuse Alto ENDS was significantly faster, similar in magnitude for the lowest Vuse Alto nicotine concentration assessed, and higher for the higher Vuse Alto nicotine concentrations. Subjective effects were generally similar between the Vuse Alto ENDS and NRT gum, although in some instances subjective effects such as liking of positive effects were significantly higher. Taken together, our results indicate that the AL of the Vuse Alto ENDS falls between that of cigarettes and NRT gum, even at the highest (5%) nicotine concentration. Of further note, reductions in urge to smoke associated with the use of Vuse Alto ENDS was comparable to that of NRT gum use, although both products did not reduce smoking urges as much as cigarettes. The reduced AUC_0-15_ for the Vuse Alto ENDS represents faster and higher nicotine delivery, in a manner similar to combustible cigarettes, an important factor associated with continued product use, interest in using the products in the future, and the product’s AL (Carter et al., 2009; Henningfield et al., 2011). This suggests that Vuse Alto ENDS deliver nicotine at a sufficient rate, which may help smokers transition away from smoking. Overall, and in their totality, these findings suggest that Vuse Alto ENDS may provide a satisfying and acceptable form of nicotine delivery for cigarettes smokers, while not having such as high AL as to pose an addiction and/or initiation risk greater than cigarettes, and is suggestive of a potential positive role for Vuse Alto ENDS in THR.

A key factor attributed to the popularity of ENDS use and a subject of debate is flavors (Gades et al., 2022). Some studies have reported preferences for sweet and menthol flavors among various types of ENDS users (Rostron et al., 2020; Soneji et al., 2019), and concerns over the use of flavored e-cigarettes by youth have been raised (Cooper et al., 2022; Park-Lee et al., 2022). Data from these studies can also be used to explore the potential impact of flavors on nicotine uptake and product appeal. Seven different flavors as well as an Unflavored Vuse Alto ENDS, each with the same nicotine concentration (1.5%), were assessed in Study 1, while two different flavors with the same nicotine concentration (1.5%) were assessed in Study 2. In each study, the nicotine PK profiles, both in terms of the rate and the magnitude of nicotine delivery, were similar across the different flavor variants assessed. Furthermore, comparisons across studies also suggest broadly similar nicotine delivery across the flavor variants evaluated. Additionally, subjective effects observed in both Studies 1 and 2 were generally similar. However, it should be noted that emerging evidence indicates that certain flavoring constituents may independently influence toxicological profiles and AL, beyond nicotine effects alone. For example, flavoring ingredients commonly used in ENDS e-liquids have been shown to induce cytotoxicity, oxidative stress, and inflammatory responses in vitro, and may contribute to the formation of aerosol toxicants during heating (Salam et al., 2020; Muthumalage et al., 2018). Additionally, the availability and type of flavors may influence product appeal and AL, with evidence suggesting that flavor variety can increase product attractiveness and potentially reinforce use (Jabba et al., 2020; Leigh et al., 2016). These aspects were not specifically evaluated in the present studies and warrant further investigation when interpreting the potential health and behavioral impacts of flavored ENDS products.

Overall, these findings suggest that the AL of the Vuse Alto ENDS tested is similar regardless of the flavor when the nicotine concentration remains the same. This is in line with previous studies which have demonstrated no major differences in nicotine PK and product liking between tobacco and other flavor variants of Vuse Solo ENDS with the same nicotine concentration (Keyser et al., 2023), and no major differences between nicotine PK and subjective effects between menthol flavors (Stiles et al., 2017) and among tobacco-flavored variants (Stiles et al., 2017) of Vuse Solo ENDS with the same nicotine concentration. Furthermore, a number of other clinical studies have supported the hypothesis that flavor does not impact nicotine PK or AL of both open- and closed-system ENDS (Goldenson et al., 2020a; Fearon et al., 2022; Barnes et al., 2017; Cobb et al., 2019; St Helen et al., 2017), with support for this hypothesis also coming from survey studies (Buu et al., 2018).

Nicotine delivery and uptake has also been reported as dependent on design features and nicotine concentrations of ENDS (DeVito and E-cigarettes, 2018; Foulds et al., 2015; Harvanko et al., 2018; Martínez et al., 2020). In Study 2, there was a slight increase in nicotine uptake with the higher e-liquid nicotine concentrations. This is consistent with previous work, although the relationship has not always been found to be linear (Goldenson et al., 2020b; Goldenson et al., 2022; Goldenson et al., 2021; Phillips-Waller et al., 2021; Walele et al., 2016). While the use of ENDS with higher concentrations of nicotine may be associated with higher AL, the higher degree of nicotine delivery from such ENDS may help them serve as better substitutes of cigarettes and facilitate complete substitution for cigarettes (Gades et al., 2022; Goldenson et al., 2022; Goldenson et al., 2021; Phillips-Waller et al., 2021; Hajek et al., 2020; Carpenter et al., 2017), as has been reported for other nicotine-containing products such as gums and patches (Lindson et al., 2019; Tobacco Use and Dependence Guideline Panel, 2008; Stead et al., 2008).

Studies 1 and 2 of Vuse Alto ENDS discussed in this paper have several strengths and some limitations. First, both studies were conducted in clinical confinement using the well-established methodologies to assess nicotine PK and AL (Carter et al., 2009; Fearon, 2022; Vansickel et al., 2022), and were aligned with FDA guidance (U.S. Food and Drug Administration, 2021). Second, the AL of Vuse Alto ENDS was comparatively assessed relative to high and low AL comparators in Study 2. However, a potential weakness of Study 1 is that while it evaluated eight Vuse Alto ENDS flavor variants and OPL, it did not assess any nicotine-containing comparator product. However, the PK profiles and the PK metrics of the ENDS products evaluated were similar to those assessed in Study 2, in which high and low AL comparators were assessed. Further, the PK metrics and the AL of ENDS, relative to cigarettes and NRT gum were well-characterized previously by us (Stiles et al., 2017; Stiles et al., 2018; Campbell et al., 2023; Campbell et al., 2022) and others (Goldenson et al., 2020a; Maloney et al., 2019; Goldenson et al., 2020b; Goldenson et al., 2021; O'Connell et al., 2019) across several market comparators. Studies 1 and 2 reported here were potentially limited by the short periods in which participants were able to familiarize themselves with the products and acclimate to their use. Since it has been previously demonstrated that nicotine delivery from ENDS may be greater among users who are more accustomed to using them compared with naïve ENDS users, and considering that full acclimation may take many weeks (Farsalinos et al., 2015; Hajek et al., 2015), the AL may be different for the Vuse Alto ENDS following a more prolonged period of use. Despite this, and given previously published data (Stiles et al., 2017; Stiles et al., 2018; Campbell et al., 2023; Campbell et al., 2022; Goldenson et al., 2020b; Goldenson et al., 2021; Phillips-Waller et al., 2021), it is not anticipated that this would change the relative AL of Vuse Alto ENDS compared with cigarettes and NRT, or the lack of a flavor impact on AL. Finally, a study limitation related to gender distribution should be acknowledged, as enrollment did not meet the prespecified target of ≥40% representation for females (although both studies met the male enrollment target). In Study 1, females comprised 26% (10/38) of participants and males 74% (28/38), while in Study 2, females comprised 33% (14/43) and males 67% (29/43). This imbalance may limit the generalizability of the findings, particularly given reported gender-related differences in nicotine pharmacokinetics and subjective responses (e.g., faster nicotine metabolism in females and greater dose sensitivity in males), although findings are variable across studies (Benowitz et al., 2006; Perkins et al., 2018; Streck et al., 2020).

In summary, the findings from Study 1 found that tobacco and non-tobacco flavor variants of Vuse Alto ENDS showed similar nicotine uptake and similar scores for OPL. In Study 2, nicotine delivery was dependent on the nicotine concentration of Vuse Alto ENDS, but both nicotine delivery and overall AL were not influenced by Vuse Alto ENDS flavor. The AL of the Vuse Alto ENDS is lower than that of cigarettes but equal to or greater than that of NRT gum.

In terms of the overall potential public health benefit, our findings support that Vuse Alto ENDS may provide a potentially viable and acceptable alternative to cigarette smoking for adult smokers, based on lower toxicant emissions and intermediate AL profiles relative to cigarettes. These characteristics Vuse Alto ENDS may support a role for Vuse Alto ENDS within a harm reduction framework by facilitating transition away from smoking among adult smokers. However, these potential benefits must be considered alongside important limitations observed at the population level, including the prevalence of dual use (concurrent use of ENDS and cigarettes), which may attenuate reductions in toxicant exposure, as well as the potential for initiation of nicotine use among individuals who do not currently use tobacco products. Accordingly, while Vuse Alto ENDS may contribute to harm reduction for some adult smokers, their overall public health impact depends on patterns of use, including complete switching versus dual use and uptake among non-users of tobacco.

## Data Availability

The original contributions presented in the study are included in the article/Supplementary Material, further inquiries can be directed to the corresponding author.
