# Supplementary material for: Assessment of nicotine pharmacokinetics and abuse liability in randomized, crossover studies of Vuse Alto electronic nicotine delivery systems
Source: Front Pharmacol. 2026 Jul 14;17:1796858. doi: 10.3389/fphar.2026.1796858 (PMC13408391; doi:10.3389/fphar.2026.1796858)
Supplement: Supplementary file 1 [file DataSheet1.pdf]

Supplementary Material

1 Supplementary Tables

Supplementary Table 1: Disposition of study participants in Study 1 and Study 2

	**Study 1**	**Study 2**
Participants Enrolled (n)	38	43
Participants Randomized (n)	38	43
Participants Completed (n [%])	36 (95)	42 (97.7)
Participants Discontinued (n [%])	2 (5)	1 (2.3)
Reason for Study Discontinuation (n [%])		
Non-compliance with study product use	1 (2.3)	0
Physician decision	1 (2.3)	1 (3.0)

Abbbreviations: n, number of observations.

Supplementary Table 2: Demographics and baseline characteristics of study participants in Study 1 and Study 2

Demographic/Characteristic	Study 1	Study 2
Number of participants	38	43
Age (years): mean (SD)	43.2 (10.01)	39.1 (10.0)
Weight (kg): mean (SD)	87.6 (16.5)	96.5 (20.2)
Height (cm): mean (SD)	174.1 (7.5)	174 (9.97)
BMI (kg/m^2): mean (SD)	29.0 (5.3)	31.8 (6.26)
Gender [n (%)]		
Male	28 (74)	29 (67)
Female	10 (26)	14 (33)
Ethnicity [n (%)]		
Hispanic/Latino	3 (8)	0
Not Hispanic/Latino	35 (92)	43 (100.0)
Race [n (%)]		
White	13 (34)	29 (67.4)
Black/African American	21 (55)	12 (27.9)
American Indian or Alaska Native	2 (5)	1 (2.3)
Native Hawaiian or Other Pacific Islander	1 (3)	1 (2.3)
Not Reported	1 (3)	0

Abbreviations: BMI, body mass index; n, number of observations; SD, standard deviation.

Supplementary Table 3: Summary of adverse events in Study 1

	Study check-in	Vuse Alto ENDS (1.5% nicotine concentration)								
		Menthol N = 37	Rich Tobacco N = 37	Golden Tobacco N = 37	Smooth Tobacco N = 37	Aromatic Tobacco N = 36	Tropical Coconut N = 36	Berry Cream N = 36	Unflavored N = 37	Overall
Number of Participants with AEs (n [%])	1 (3)	0	0	0	0	0	0	1 (3)	1 (3)	3 (8)
Eye disorder (n)	1	0	0	0	0	0	0	0	0	1
Conjunctival irritation	1									1
General disorders & administration site conditions (n)	0	0	0	0	0	0	0	1	0	1
Vessel puncture site pain								1		1
Musculoskeletal and connective tissue disorders (n)	0	0	0	0	0	0	0	0	1	1
Musculoskeletal pain									1	1

Adverse events reported are those that were observed during clinical confinement. Adverse events were classified according to MedDRA Version 24.0. Abbreviations: AE, adverse event; ENDS, electronic nicotine delivery system(s); N, number of participants; n, number of observations.

Supplementary Table 4: Summary of severity of reported adverse events and their causal relationship to study product use in Study 1

	Vuse Alto ENDS (1.5% nicotine concentration)							
	Menthol<br>N = 37	Rich Tobacco<br>N = 37	Golden Tobacco<br>N = 37	Smooth Tobacco<br>N = 37	Aromatic Tobacco<br>N = 36	Tropical Coconut<br>N = 36	Berry Cream<br>N = 36	Unflavored<br>N = 37
Number of participants with AEs (n [%])	0	0	0	0	0	0	1 (3)	1 (3)
Any AE (n [%])								
Causally related to study product use	0	0	0	0	0	0	0	0
Possibly related to study product use	0	0	0	0	0	0	0	0
Related to study product use	0	0	0	0	0	0	0	0
Participants with AE severity (n [%])								
Mild	0	0	0	0	0	0	1 (3)	1 (3)
Moderate	0	0	0	0	0	0	0	0
Severe	0	0	0	0	0	0	0	0

Abbreviations: AE, adverse event; ENDS, electronic nicotine delivery system(s); UB, usual brand; N, number of participants; n, number of observations.

Supplementary Table 5: Summary of adverse events in Study 2

System Organ Class	Pre-test session[a]	Study Product Menthol (1.5%) N = 43	Menthol (2.4%) N = 43	Menthol (5%) N = 42	Glacier Menthol (1.5%) N = 42	UB Cigarette N = 43	NRT gum (4 mg) N = 42	Overall N = 43
Participants with any AE (n [%])	1 (2.3)	4 (9.3)	0	0	1 (2.4)	3 (7.0)	2 (4.8)	9 (20.9)[b]
Gastrointestinal disorders (n)	0	2	0	0	0	0	0	2
Toothache		2	0	0	0	0	0	2
General disorders & administration site conditions (n)	0	0	0	0	0	2	0	2
Fatigue						1		1
Malaise						1		1
Nervous system disorders (n)	1	1	0	0	0	4	1	7
Dizziness	0	1				1	1	3
Headache	1	0				2	0	3
Syncope	0	0				1	0	1
Reproductive system and breast disorders (n)	0	0	0	0	1	0	0	1
Dysmenorrhea					1			1
Respiratory, thoracic, and mediastinal disorders (n)	1	1	0	0	0	0	0	2
Throat irritation	1	0						1
Wheezing	0	1						1
Skin & subcutaneous tissue disorders (n)	0	0	0	0	0	0	1	1
Rash							1	1
Vascular disorders (n)	0	1	0	0	0	0	0	1
Orthostatic hypotension		1						1

Adverse events reported are those that were observed at check-in, during the pre-study product acclimation period, and during clinical confinement. Adverse events were classified according to MedDRA Version 24.1. Percentages in parentheses are the Vuse Alto ENDS nicotine concentrations. Abbreviations: AE, adverse event; ENDS, electronic nicotine delivery system(s); UB, usual brand; NRT, nicotine replacement therapy; N, number of participants; n, number of observations. [a]AEs that occurred prior to the start of product use in the first test session; [b]one participant reported two AEs before start of the test session (pre-test) and during test sessions for UB cigarette and NRT gum.

Supplementary Table 6: Summary of severity of reported adverse events and their causal relationship to study product use in Study 2

	Study Product					
	Menthol (1.5%) N = 43	Menthol (2.4%) N = 43	Menthol (5%) N = 42	Glacier Menthol (1.5%) N = 42	UB Cigarette N = 43	NRT gum (4 mg) N = 42
Number of participants with AEs [n (%)]	4 (9.3)	0	0	1 (2.4)	3 (7.0)	2 (4.8)
Any AE [n (%)]						
Causally related to study product use	2 (4.7)	0	0	0	0	0
Possibly related to study product use	0	0	0	0	0	0
Related to study product use	2 (4.7)	0	0	0	0	0
Participants with AE severity [n (%)]						
Mild	4 (9.3)	0	0	1 (2.4)	3 (7.0)	2 (4.8)
Moderate	0	0	0	0	1 (2.3)	0
Severe	0	0	0	0	0	0

Percentages in parentheses are the Vuse Alto ENDS nicotine concentrations. Abbreviations: AE, adverse event; ENDS, electronic nicotine delivery system(s); UB, usual brand; NRT, nicotine replacement therapy; N, number of participants; n, number of observations.
